# Mycotoxins: Toxicology, Identification and Control

**DOI:** 10.3390/toxins13040242

**Published:** 2021-03-29

**Authors:** Cristina Juan García

**Affiliations:** Laboratory of Food Chemistry and Toxicology, Faculty of Pharmacy, University of Valencia, Avda. Vicent Andrés Estellés, S/N, 46100 Burjassot-Valencia, Spain; cristina.juan@uv.es

The evaluation of the presence of mycotoxins in different matrices is achieved through different analytical tools (including quantitative or qualitative determinations). Research on optimal mycotoxins’ extraction and clean-up methods, combined with chromatographic equipment coupled to mass spectrometric detectors (Triple quadrupole/linear ion trap mass spectrometry, tandem mass spectrometry, quadrupole time-of-flight mass spectrometry) is of the utmost importance for accurate measurements of mycotoxins in diverse matrices. All these techniques and methodologies imply key steps in the establishment of the limits of detection, limits of quantification, points of identification, accuracy, reproducibility, and/or repeatability of different procedures. The maximum levels or recommended levels for mycotoxins in different matrices are comprised within a wide range (including the levels tolerated by infants and animals). In addition, their control and evaluation of exposure are demanded by authorities and food safety systems.

Food and feed authorities are concerned not only with the determination of presence of mycotoxins but also with the toxicological effects of them, and in vivo or in vitro assays are necessary for a complete evaluation. In fact, these assays are the basis for the control and prevention of population exposure to mycotoxins in dietary exposure studies. Recent surveys are focused on regulated mycotoxins (aflatoxins, fumonisins, and trichothecenes) and emerging toxins such as enniatins and beauvericin in adult consumers, while very few studies have monitored mycotoxins levels in infant products.

This Special Issue of Toxins comprises 11 original contributions and one review. The issue reports new findings regarding the presence of mycotoxins in aromatic and medicinal plants, mango and orange juice, juices, pulps, jams and beer, from Morocco, Pakistan, and Portugal. In these studies, innovative techniques to study their presence have been developed. El Jai et al. [1] used liquid chromatography coupled to time-of-flight mass spectrometry to analyse mycotoxins and conjugated mycotoxins; there were a total of 14 mycotoxins in 40 samples of aromatic medicinal plants (AMPs) from Morocco. Hussain et al. [2] analyzed patulin in 274 fruit and derived products samples from Pakistan, and Silva et al. [3] evaluated the presence of ochratoxin A in 85 beer samples from Portugal. These results revealed that regular monitoring of cereals or fruits and their products (beer, juices, pulps and jams) during the harvest and processing stages is recommended to enhance the confidence in final consumers.

The Special Issue also presents novel strategies to detect the presence of mycotoxins as reported by Efremenco et al. [4]. They compared the characteristics of a rapid quantitative analysis of different mycotoxins (deoxynivalenol, ochratoxin A, patulin, sterigmatocystin, and zearalenone) using acetyl-, butyrylcholinesterases and photobacterial strains of luminescent cells. The best bioindicators in terms of sensitivity and working range (μg/mL) were as follows: *Photobacterium* sp. 17 cells for analysis of deoxynivalenol (0.8–89) and patulin (0.2–32); *Photobacterium* sp. 9.2 cells for analysis of ochratoxin A (0.4–72) and zearalenone (0.2–32); and acetylcholinesterase for analysis of sterigmatocystin (0.12–219).

Related with this highlighted scenario, Rodríguez-Carrasco et al. [5] have evaluated the exposure to enniatin B1 by biomonitoring metabolites in urine and identifying as major products: hydroxylated metabolites (78% samples) and carbonylated metabolites (66% samples). Also toxicological effects of zearalenone metabolites and beauvericin were evaluated by Agahi et al. [6]. They evaluated the metabolism and toxicological effects on SH-SY5Y neuronal cells and IC_50_ values for the individual and combined treatments of the mentioned mycotoxins.

One important point in control of mycotoxins is decontamination strategies, and in this sense, Oliveria da Cruz et al. (2021) [7] evaluated the efficacy of potentially probiotic fruit-derived *Lactobacillus* isolates to remove aflatoxin M_1_ (AFM_1_) from a phosphate buffer solution (PBS; spiked with 0.15 µg/mL AFM_1_). The authors concluded that *L. paracasei 108, L. plantarum 49*, and *L. fermentum 111* could have potential application to reduce AFM_1_ to safe levels in foods and feeds.

Abbasi Pirouz et al. [8] present a simultaneous removal of 11 mycotoxins in palm kernel cake (PKC) using chitosan. PKC is used in ruminant feed; its use in poultry, swine, and fish diets is as a valuable source of protein and energy, while chitosan is a polyaminosaccharide and the second most abundant bio-polymers after cellulose. Mycotoxins studied were: aflatoxins (AFB_1_, AFB_2_, AFG_1_ and AFG_2_), ochratoxin A (OTA), zearalenone (ZEN), fumonisins (FB_1_ and FB_2_), trichothecenes (deoxynivalenol (DON), HT-2, and T-2 toxin) [8].

Another mycotoxin decontamination technique was tested by Yang et al. [9]. They used electron beam irradiation (EBI) and ozone on the degradation of ZEN and OTA. It was observed that 2 mL of 50 µg/mL of ZEN and OTA was completely reduced for ZEN and when 50 mg/L ozone is used, OTA is reduced at 34%. Acetamiprid was used by Nowak et al. [10] to reduce the production of destruxins produced by *Metarhizium* sp. Acetamiprid at concentrations from 5–50 mg/L did not inhibit the growth of all tested *Metarhizium* sp.; however, it reduced the level of 19 produced destruxins in direct proportion to the dosage used. Also, Wang et al. [11] studied a destruxin mycotoxin: destruxin A (DA), a cyclodepsipeptidic mycotoxin with pesticide proprieties involved in regulation of transcription and protein synthesis. It was suggested that silkworms’ arginine tRNA synthetase (BmArgRS), Lamin-C Proteins (BmLamin-C), and ATP-dependent RNA helicase PRP1 (BmPRP1) were candidates of DA-binding proteins.

Finally, this Special Issue includes a review by Zhu et al. [12], which summarizes the newly discovered macrocyclic trichothecenes and their bioactivities over the last decade, as well as identifications of genes tri17 and tri18 involved in the trichothecene biosynthesis and putative biosynthetic pathway [12].
